# Inhibition of interferon signaling improves rabbit calicivirus replication in biliary organoid cultures

**DOI:** 10.1128/jvi.00574-25

**Published:** 2025-07-25

**Authors:** Elena Smertina, Megan Pavy, Nias Y. G. Peng, Omid Fahri, Maria Jenckel, Tanja Strive, Michael Frese, Ina L. Smith

**Affiliations:** 1 Commonwealth Scientific and Industrial Research Organisation, Health and Biosecurity, Black Mountain 2221, Canberra, Australian Capital Territory, Australia; 2 Faculty of Science and Technology, University of Canberra 2234, Canberra, Australian Capital Territory, Australia; University Medical Center Freiburg, Freiburg, Germany

**Keywords:** RHDV, RHDV2, Ruxolitinib, rabbit, liver organoids

## Abstract

The *Rabbit hemorrhagic disease virus* (RHDV) was discovered 40 years ago. This highly pathogenic virus threatens the integrity of ecosystems in the European rabbit’s native range, while in Australia, it is used as a biocontrol tool to manage overabundant populations of feral European rabbits. Little is known about the life cycle of this virus due to the absence of a reliable cell culture system. In 2023, we developed a rabbit liver-derived organoid cell culture system that supports RHDV replication but is unable to sustain serial passaging in culture. Here, we report that the interferon signaling pathway inhibitor Ruxolitinib increases virus replication in organoid-derived monolayer cells and, for the first time, enables the serial passaging of RHDV in cell culture. Four consecutive passages were achieved with viral titers reaching the concentration of the initial virus stock as measured by real-time quantitative PCR. Immunofluorescence analysis showed that more cells are infected in the presence of Ruxolitinib. Furthermore, we noted that cells grew faster and formed healthier monolayers in the presence of the interferon inhibitor. To determine the cellular composition of the monolayers, we used single-cell RNA sequencing, revealing that our organoids consist largely of RHDV-permissive cholangiocytes.

**IMPORTANCE:**

In this work, we describe the use of an interferon inhibitor to enhance the permissiveness of our recently developed rabbit liver-derived organoid cell culture system for rabbit hemorrhagic disease viruses. The data show that interferon inhibitors offer a simple and cost-effective approach to increase the replication of difficult-to-grow viruses in culture systems that are not interferon-deficient.

## INTRODUCTION

Rabbit hemorrhagic disease viruses (RHDVs) are highly pathogenic, positive-sense, single-stranded RNA viruses in the genus *Lagovirus*, family *Caliciviridae*, that infect lagomorphs with the European rabbit (*Oryctolagus cuniculus*) as the main host species. There are two major genotypes: RHDV1 infects only rabbits, while RHDV2 also infects other lagomorphs, such as hares. Both genotypes are hepatotropic, and an infection usually leads to peracute liver disease, characterized by necrosis, hemorrhages, and death within 72 h. The highly virulent nature makes RHDV an efficient biocontrol agent for introduced European rabbit populations in Australia and New Zealand (reviewed in reference 1).

Although RHDV1 was discovered in 1984 (2) and has been used in Australia as a biocontrol agent since the mid-1990s, knowledge of many aspects of the viral life cycle is limited. For example, the virus entry receptor is unknown, and the functions of several of its non-structural proteins have not yet been determined. This can mainly be attributed to the inability to efficiently grow rabbit caliciviruses in cell culture, a problem that is frequently encountered with hepatotropic viruses, such as the human hepatitis B and C viruses (reviewed in reference 3). Hepatocytes, the main target cells for these viruses, are highly differentiated cells that are difficult to grow and maintain in cell culture. Recently, we established a rabbit liver-derived organoid culture system that supports the replication of both RHDV1 and RHDV2 (4). However, only a small proportion of organoid cells produced detectable amounts of viral antigen, and attempts to passage the virus in liver organoids were not successful. Thus, further improvements were needed to generate a more robust cell culture system.

Virus infections in cell culture often trigger interferon (IFN) responses mediated by IFN type I (IFN-α, IFN-β, and others) and IFN type III (IFN-λ); IFN type II (IFN-γ) is usually not a factor that limits virus replication in cell culture as it is predominantly produced by immune cells (reviewed in reference 5). In immune-competent cells, the production of IFN and the subsequent expression of IFN-induced genes can inhibit or completely block virus multiplication. For example, human noroviruses (family *Caliciviridae*) induce innate immune responses in human intestinal organoids via IFN types I and III (6, 7). As IFNs regulate gene expression through the Janus kinase (JAK) signal transducer and activator of transcription (STAT) signaling pathway (reviewed in reference 8), it is not surprizing that genetically engineered STAT1-knockout intestinal organoids demonstrate an increased permissiveness to human norovirus infection (7).

Ruxolitinib (Rux), a small-molecule JAK1 and JAK2 inhibitor, inhibits early stages of all types of IFN signaling pathways by competing with adenosine triphosphate (ATP) for the ATP-binding pocket of these kinases (9). Rux and similar IFN inhibitors increase the replication of a range of viruses when used as a supplement in cell culture media (6, 10–12). Apart from inhibiting IFN-mediated responses, Rux can induce apoptosis in cancer cells (13, 14), which makes it difficult to reliably predict the benefit of its use in cell culture.

With this work, we aimed to optimize the rabbit liver-derived organoid system to enable passaging of lagoviruses and the production of high-titered virus stocks by assessing the ability of the IFN signaling inhibitor Rux to increase RHDV replication. Furthermore, using single-cell RNA sequencing, we determined the cellular composition of the organoid-derived monolayers, which revealed that our organoids contain predominantly cholangiocytes.

## MATERIALS AND METHODS

### Establishment and culturing of rabbit liver organoids and organoid-derived monolayers

The rabbit organoid (spheroid) cultures and organoid-derived monolayers were established and passaged as described previously (4). Modifications included the use of Rux (Selleckchem or Sapphire Bioscience) in cell culture media (with a concentration of 4 µM unless otherwise specified) starting from the first seeding of isolated stem cells unless otherwise specified. Rux stock solution was prepared by reconstitution in dimethyl sulfoxide (DMSO) to a 10 mM concentration.

### Viruses

RHDV1 (genotype GI.1cP-GI.1c according to Le Pendu et al. [15]; GenBank accession number KT344772) and RHDV2 (genotype GI.1bP-GI.2; GenBank accession number MW467791) stocks were prepared by the Elizabeth Macarthur Agricultural Institute (Menangle, NSW, Australia) from semi-purified liver homogenates after amplification in domestic rabbits, and the rabbit infectious dose (ID_50_) was titrated in rabbits as described previously (16, 17). Freeze-dried virus stocks were reconstituted in unsupplemented Advanced DMEM/F12 (Sigma-Aldrich) and stored at −80°C.

### Inoculation of organoid-derived monolayer cells

The organoid-derived monolayers were seeded on Geltrex (Gibco)- or Matrigel (Corning)-coated surfaces. The coating was performed using a 2% solution of Geltrex or Matrigel in Dulbecco’s phosphate-buffered saline (DPBS; Sigma-Aldrich) for 2 h at 37°C. The seeding density had been determined empirically and is 80,000 cells per well for an 8-well chamber slide (Thermo Scientific). The cells were inoculated in triplicates with either RHDV1 or RHDV2 at a multiplicity of infection (MOI) of 0.01. The inoculum was diluted in DMEM high glucose medium (Sigma-Aldrich) supplemented with 2% heat-inactivated fetal calf serum (FCS) (Thermo Scientific). Mock-infected cells and cells incubated with heat-inactivated RHDV1 and RHDV2 (65°C for 15 min) were used as controls. After 1 h at 37°C in a 5% CO_2_ incubator, the inoculum was removed, and cells were washed with DPBS three times. Fresh culture medium (composition as described previously [4]) was added, and the cells were incubated for 1, 48, or 72 h, as specified.

### Cytotoxicity assay

Cytotoxicity of Rux in organoid-derived monolayer cells was measured using the CyQUANT MTT Cell Proliferation Assay kit (Thermo Scientific) (18) according to the manufacturer’s instructions for the rapid protocol. Briefly, monolayer cells were seeded at 10,000 cells per well in a 96-well plate and cultured in phenol red-free media overnight. Rux was added at 1, 4, 8, or 16 µM; unsupplemented and media supplemented with DMSO equivalent to a 16-µM Rux treatment were used as negative controls. The apoptosis inducer Camptothecin (Sigma-Aldrich) was used as a positive control at 20 µM. After the treatment, monolayers were incubated for 48 h before plates were analyzed by measuring the absorbance at 540 nm with a FLUOstar Omega plate reader (BMG Labtech). The absorbance of “blank” wells without cells was measured, and the background signal was subtracted from all other measurements using the MARS software (BMG Labtech).

### RNA extraction and RT-qPCR

Total RNA was extracted using the NucleoSpin RNA Mini kit (Macherey-Nagel) or the KingFisher MagMAX Viral/Pathogen Nucleic Acid Isolation kit (Applied Biosystems) on the KingFisher Flex instrument (Thermo Scientific) according to the manufacturers’ instructions, and viral RNA was quantified using a universal real-time quantitative PCR (RT-qPCR) assay targeting a conserved region of the lagovirus VP60 coding sequence as described previously (17) using the SensiFAST SYBR No-ROX kit (Bioline Reagents, Meridian Bioscience) on a CFX96 Touch Real-Time PCR instrument (Bio-Rad). A standard curve was utilized for absolute quantification; “no template,” a previously quantified positive control, and extraction controls were included in each analysis. Each sample was processed using three technical replicates. The data were analyzed with CFX Maestro Bio-Rad software, and results reported as log_10_ capsid gene copies per well.

To analyze IFN-induced gene expression, primers were designed to cover intron-spanning regions using Primer Express 2.0 (Thermo Scientific) or PrimerQuest Tool (Integrated DNA Technologies) software (Table S1). RT-qPCRs were performed using the SensiFAST Probe No­-ROX kit (Bioline Reagents, Meridian Bioscience) on a CFX96 Touch Real-­Time PCR Instrument (Bio­-Rad) as follows: reverse transcription at 45°C for 10 min, followed by initial denaturation at 95°C for 2 min, and then 40 cycles of 95°C for 5 s and 60°C for 20 s. A “no template“ control was added to each run. Expression of the gene of interest was normalized to expression levels of the housekeeping gene glyceraldehyde­ 3-­phosphate dehydrogenase (*GAPDH*). Fold change was calculated using the ΔC_T_ method (19).

### Immunofluorescence staining and analysis

Monolayer cells grown in chamber slides were fixed with 100% ice-cold acetone at −20°C for 12 min, washed twice with DPBS, permeabilized with 0.25% Triton X-100 (Sigma-Aldrich) in DPBS for 10 min at room temperature, washed with DPBS three times, and blocked with DPBS containing 5% bovine serum albumin (BSA) and 0.1% Tween-20 (DPBST) for 60 min. For virus capsid staining, the lagovirus-specific anti-VP60 mouse monoclonal antibody 13C10 (25 µg/mL; Monoclonal Antibody Facility of the Institute for Medical Research, Perth, Australia) (20) was used at a 1:100 dilution in 1% BSA in DPBST. The slides were then incubated overnight at 4°C. The cells were then washed three times with DPBS, and secondary antibodies conjugated to Alexa Fluor 488 (Abcam; ab150113) were added (diluted at 1:300 in 1% BSA in DPBST). The slides were incubated at room temperature for 60 min in the dark, washed three times with DPBS, and stained with 4′,6-diamidino-2-phenylindole (DAPI) (1 mg/mL; Sigma-Aldrich) diluted at 1:500 in sterile water. The cover glass was mounted with FluoroShield (Sigma-Aldrich). Fluorescence images were obtained using a Zeiss AxioImager M2 microscope equipped with a Colibri 7 LED illumination system, a Plan-Apochromat 10× NA = 0.45 objective, and a Zeiss Axiocam 712 color CCD camera or a Zeiss Axiovert 5 microscope equipped with a Colibri 3 LED illumination system, a Plan-Neofluar 40× NA = 0.75 objective, and an Axiocam 305 mono camera (Carl Zeiss). Image capture was carried out using ZEN blue v3.2 (Carl Zeiss).

Quantitative image analysis was completed using FIJI/ImageJ v1.54 (21). Briefly, fluorescence images were converted to 8-bit grayscale, an identical threshold applied, and the FIJI “Analyze particles” function was used to quantify the total number of cells and the number of VP60-expressing cells.

### Data visualization and statistical analyses

The quantitative data were processed and visualized in R (22) using packages ggplot2 (v3.5.1) (23), patchwork (v1.3.0) (24), ggpattern (v1.1.4) (25), dplyr (v1.1.4) (26), stringr (v1.5.1) (26), rstatix (v0.7.2) (27), magrittr (v2.0.3) (28), and svglite (v2.1.3) (29). Statistical differences were assessed using one-way or two-way analysis of variance (ANOVA) (as specified) followed by Tukey’s honestly significant difference (HSD) test.

### Single-cell RNA sequencing

Organoid-derived monolayer cells were seeded on Geltrex-precoated 6-well plates using media supplemented with Rux and incubated overnight. The next day, the cells were detached using TrypLE (Sigma-Aldrich), counted using a hemocytometer, and passed through a 40-µm cell strainer. The cells were resuspended in 2% FCS in DPBS at 1 million/mL. Prior to fluorescence-activated cell sorting (FACS), propidium iodide (50 µg/mL; BD Biosciences) was added (0.1 µg/million cells) to discriminate between live and dead cells. The Hoechst 33342 (BD Biosciences) dye was used at 4 µg/mL for sorting single cells and excluding duplets and clumps. The cells were then incubated for 15 min at 37℃ prior to sorting with the FACSAria Fusion (BD Biosciences) instrument (John Curtin School of Medical Research, Australian National University (ANU), Canberra, Australia). A total of 84,000 live single cells were collected and processed using the 10x Genomics Chromium single-cell sequencing library preparation kit 3′ Next GEM v3.1 following the manufacturer’s protocol. The resulting cDNA was sequenced with Illumina NextSeq 2000 (P2 100 cycle flow cell) instrument. The sequencing and library preparation were performed by the Biomolecular Resource Facility, a National Collaborative Research Infrastructure Strategy-supported Bioplatforms Australia genomics node at the John Curtin School of Medical Research (ANU, Canberra, Australia). The raw reads were processed using Cell Ranger software (v7.1.0) (30). The reads were mapped to the *O. cuniculus* genome (National Center for Biotechnology Information RefSeq GCF_009806435.1). The marker gene expression analysis and visualization were performed with the Python package scanpy (v1.10.4) (31).

### Sequencing of passaged RHDV

RNA extracted from stock viruses (RHDV1 and RHDV2) and from the supernatants of each passage (four passages with three biological replicates for each; a total of 26 samples) was used for library preparation with the NEBNext Single Cell/Low Input RNA kit according to the manufacturer’s instructions (New England Biolabs). The library quality was assessed with TapeStation HS D1000 tapes (Agilent). Paired-end sequencing was performed with an Illumina NovaSeq X instrument (10B flow cell lane, 2 × 150 bp) at the Australian Genome Research Facility (Melbourne, Australia).

Sequence read quality was assessed and sequences were trimmed and merged using the fastp software (v0.23.2) (32). RHDV1 and RHDV2 genome sequences obtained from the respective inocula were used as references to map sequences of passaged viruses using bowtie2 (v2.5.4) (33); the bcftools software (v1.20) (34) was used for variant calling, and base frequencies at each position were quantified with the samtools (v1.18) mpileup function (34). The virus variants were analyzed and visualized using R (22) packages seqLogo (v1.72.0) (35) and seqinr (v4.2-36) (36). Sequence alignments were performed with the MAFFT algorithm (37) in Geneious Prime (v2025.1.1). Discovery Studio was used for protein structure visualization (v25.1.0.2428) (38). The crystal structure of the RHDV VP60 protruding domain dimer was obtained from Protein Data Bank, accession number 4X1X.

## RESULTS

### Rabbit liver organoid-derived monolayers are predominantly of biliary origin

**Fig 1:**
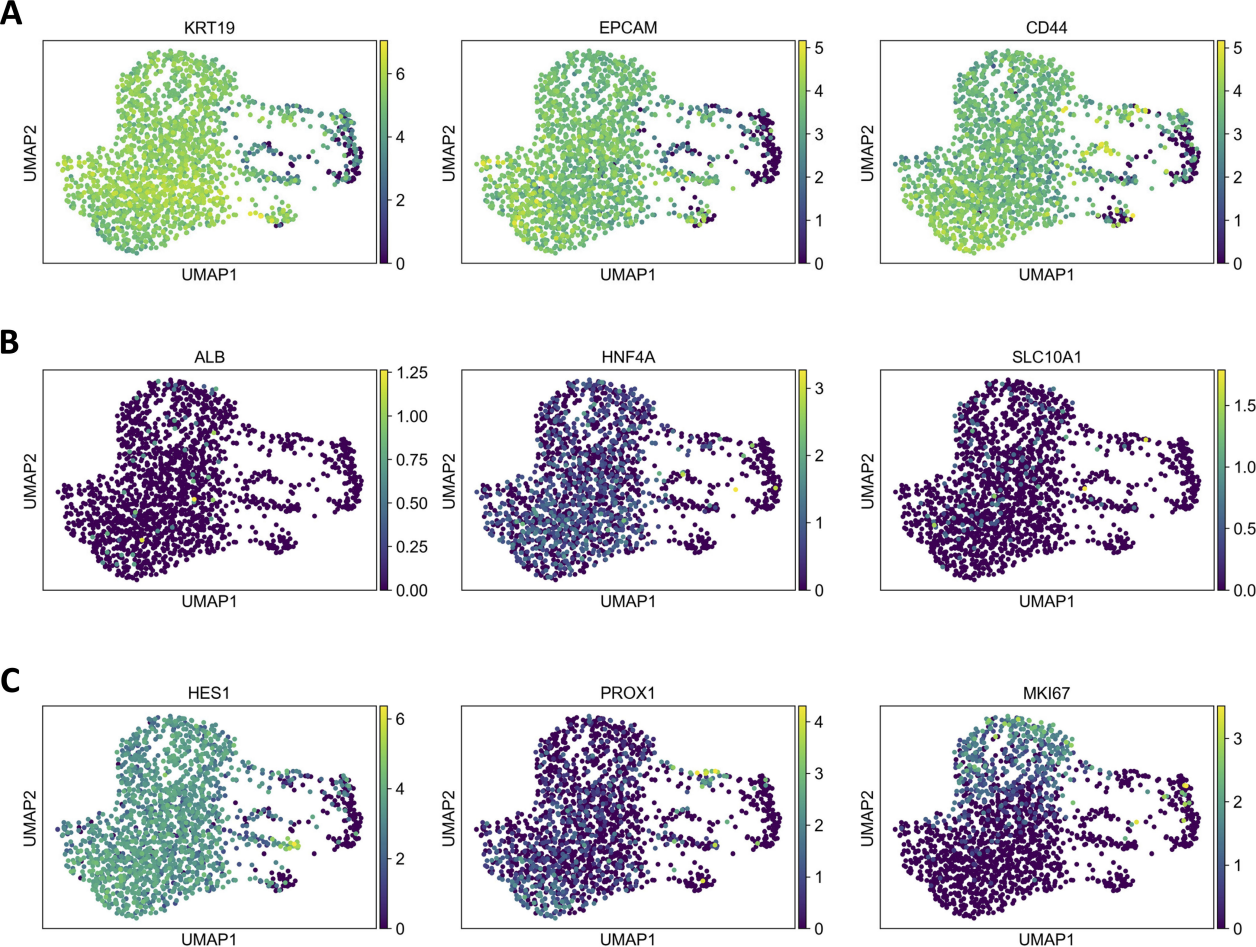
Uniform manifold approximation and projection (UMAP) plots of normalized marker gene expression in monolayer cells. Each dot represents a cell, and the color indicates the level of gene expression, with lighter colors indicating higher level of expression. (**A**) Expression of the cholangiocyte-specific markers cytokeratin-19 (KRT19), epithelial cell adhesion molecule (EPCAM), and epithelial cell marker cluster of differentiation 4 (CD44). (**B**) Expression of the hepatocyte-specific markers albumin (ALB), hepatocyte nuclear factor 4 alpha (HNF4A), and sodium-taurocholate co-transporting polypeptide (SLC10A1). (**C**) Expression of the early development transcription factor hairy and enhancer of split-1 (HES1), Prospero homeobox protein 1 (PROX1), and marker of proliferation Kiel 67 (MKI67).

Previous analyses using RT-qPCR and immunofluorescence staining revealed that the majority of the rabbit liver organoid-derived monolayer cells (hereafter referred to as “organoid-derived monolayer cells” or “monolayer cells”) are not hepatocytes (4). Although our earlier work suggested that the cells were of biliary origin, a precise analysis was challenging due to the lack of rabbit cell marker-specific antibodies. Here, to identify the cellular composition of the monolayer cells with greater precision, we used single-cell RNA sequencing (Fig. 1).

We observed increased levels of expression of cholangiocyte-specific genes compared to hepatocyte markers, in line with our previous results (4). For example, epithelial cell adhesion molecule (EPCAM) and cytokeratine-19 (KRT190 were highly expressed (Fig. 1A), a characteristic feature of cholangiocytes (39). High expression of the epithelial progenitor cell marker CD44 was also observed, suggesting that the analyzed cells are immature cholangiocytes. Mature hepatocyte markers (ALB, HNF4A, and SLC10A1) were expressed at low levels (Fig. 1B), confirming that the monolayer cells are predominantly immature bile duct cells. The conclusion was further corroborated by the high expression of the early development transcription factor HES1, which is essential for tubular bile duct formation (40), and low expression levels of PROX1, responsible for hepatocyte proliferation and migration (41).

We observed a mostly uniform expression of cell-type specific markers in our monolayer cells with the exception of the proliferation marker (MKI67) (Fig. 1C). Only a subset of cells expressed high levels of MKI67, which can be a consequence of different cell cycle stages.

### Rux enhances RHDV2 replication in organoid-derived monolayer cells

**Fig 2:**
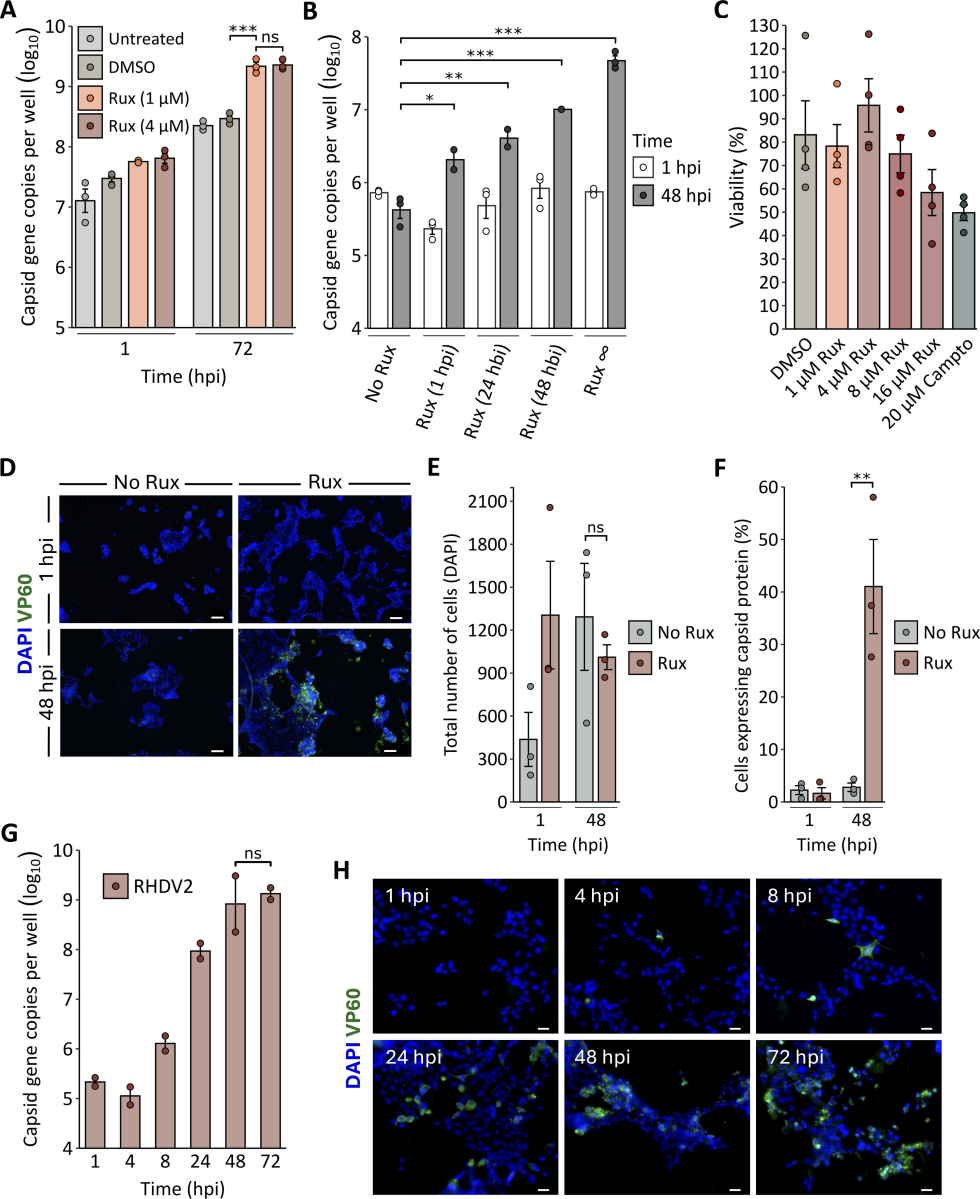
Effect of Rux on RHDV2 replication in organoid-derived monolayers. (**A**) Monolayer cells were supplemented with Rux 48 h prior to infection with RHDV2 at a MOI of 0.01. Virus replication was quantified using RT-qPCR at 1 and 72 hpi. (**B**) Rux was added to the media either before infection with RHDV2 (∞, 48, 24 hbi), or after infection (1 hpi). Virus replication was quantified using RT-qPCR at 1 and 48 hpi. Individual data points represent three biological replicates averaged from three technical replicates for each. (**C**) A tetrazolium dye (MTT) cytotoxicity assay was performed in monolayer cells after a 48 h incubation with Rux (1–16 µM, concentrations as indicated). Camptothecin (Campto) was used as a positive control, and untreated cells were used to define 100% viability. (**D**) Immunofluorescence staining of RHDV2-infected monolayer cells in the presence or absence of 4 µM Rux. Monolayer cells grown in chamber slides were stained with antibodies against the lagovirus capsid protein (VP60, green). The nuclei were counterstained with DAPI (blue). Scale bar, 100 µm. (**E**) Cells were counted based on DAPI fluorescence. (**F**) VP60 expression was quantified as a ratio of VP60-positive cells to the total number of cells. Individual data points represent three biological replicates (**E and F**). (**G** and **H**) Monolayer cells were supplemented with 4 µM Rux 48 h prior to infection with RHDV2 (MOI of 0.01). Virus replication was analyzed at 1, 4, 8, 24, 48, and 72 hpi using RT-qPCR (G) and immunofluorescence staining (H). Scale bar, 20 µm. To enhance clarity and ease of comparison, standardized scale bars were added to all immunofluorescence images. All columns and error bars represent the mean values and the standard error of the mean, respectively. Significance is denoted by asterisks (ns, not significant; *P < 0.05, **P < 0.01, ***P < 0.001) and was calculated using one-way (**A, B, and G**) or two-way (**E and F**) ANOVA followed by Tukey’s HSD test.

The small-molecule IFN inhibitor Rux was tested for its ability to boost RHDV2 replication. Rux is a pan-IFN inhibitor that affects the phosphorylation of STAT1, a key component of the IFN-induced signaling cascade. In a first experiment, organoid-derived monolayers were treated with Rux for 48 h prior to infection, and the inhibitor was tested at two concentrations, 1 and 4 µM. Cells grown in Rux-free media or media supplemented with the equivalent volume of DMSO were used as controls. Both the cells and supernatant were collected at 1 and 72 h post-infection (hpi), and virus replication was quantified using RT-qPCR. Similar (~1 log_10_) increases in RHDV2 replication were observed in cells cultured with 1 and 4 µM Rux compared to untreated or DMSO-supplemented cells at 72 hpi (Fig. 2A).

To characterize the effect of Rux over time and determine the optimal supplementation time, we investigated whether a prolonged culturing of cells in the presence of Rux (ahead of the infection) would boost virus growth further compared to shorter incubation periods. To this end, 4 µM Rux was added to the culture media either from the first cell passage of freshly cultivated cells, 48 or 24 h prior to infection, or 1 h after RHDV2 inoculation. For each group, cells that received the corresponding Rux treatment but were not infected with RHDV2 served as mock controls (not shown). A significant increase in virus replication was detected in all treatment groups compared to cells that did not receive Rux (Fig. 2B). Furthermore, we noted that a prolonged culturing of cells in the presence of Rux tended to result in higher virus replication (Fig. 2B). No statistically significant cytotoxic effect of Rux was observed or measured at all concentrations tested (Fig. 2C).

Next, we analyzed the effect of Rux at a cellular level by immunofluorescence staining of RHDV2-infected cells cultured with or without 4 µM Rux. A significant increase in the number of RHDV2-infected cells cultured with Rux was detected at 48 hpi (Fig. 2D and F). Although an equal cell number was seeded in each well, more cells were observed in Rux-supplemented samples at 1 hpi compared to Rux-free wells (Fig. 2D and E), suggesting that an incubation with Rux promotes the growth of monolayer cells. Of note, cell numbers at 48 hpi were similar in cultures with or without Rux (Fig. 2E), indicating that the difference in the number of VP60 (capsid) expressing cells cannot be attributed to the difference in the total cell number at that timepoint (Fig. 2F). Rather, the observed difference is due to a reduced permissiveness and/or a lower number of cells at 1 hpi in Rux-free samples.

To study RHDV2 replication kinetics and to determine the optimal point in time for analyzing virus yields, a time course experiment was performed. Virus genome copy numbers detected in the supernatant rapidly rose within 24 hpi and plateaued around 48 hpi with no significant difference in copy number detected between 48 and 72 hpi (Fig. 2G). Cells expressing the virus capsid protein were detected as early as 4 hpi (Fig. 2H).

### Rux inhibits the expression of IFN-induced genes in infected organoid-derived monolayer cells

**Fig 3:**
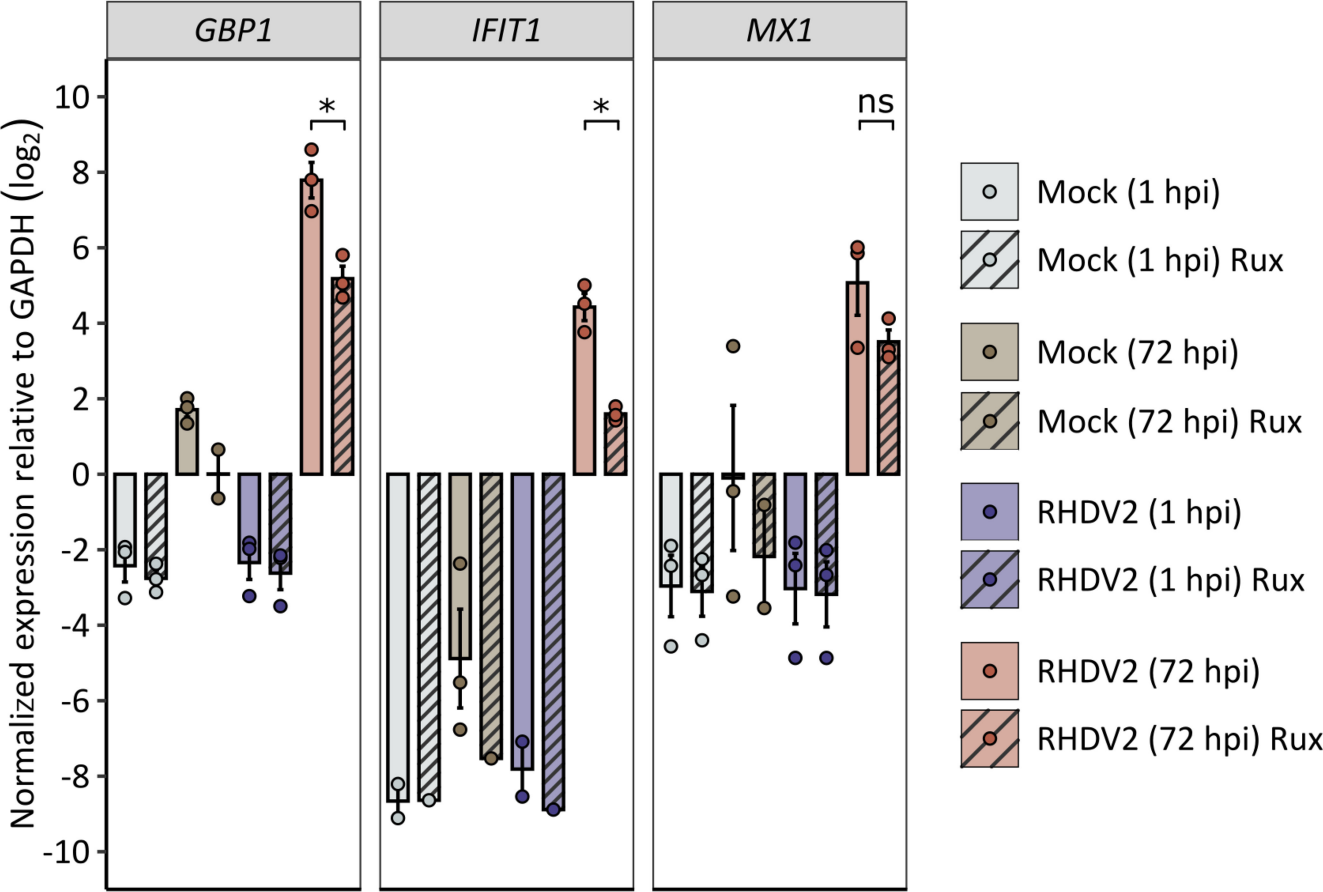
Quantification of IFN-induced gene expression in monolayer cells. Expression of *GBP1*, *IFIT1*, and *MX1* was measured using RT-qPCR in mock-infected (gray and brown) and RHDV2-infected (purple and red) monolayer cells at 1 and 72 hpi. Striping indicates the addition of 4 µM Rux 48 h prior to infection. Gene expression is presented as log_2_ fold change (ΔC_T_) relative to the expression of a housekeeping gene (*GAPDH*). Means were calculated from three independent biological replicates with three technical RT-qPCR replicates each. Error bars represent the standard error of the mean. Asterisks indicate statistical significance (ns, not significant; *P < 0.05), as determined by one-way ANOVA followed by Tukey’s HSD test.

To investigate the effect of Rux on IFN signaling in RHDV-infected cells, we quantified the expression of three IFN-induced genes, that is, guanylate-binding protein 1 (*GBP1*), IFN-induced protein with tetratricopeptide repeats 1 (*IFIT1*), and myxovirus resistance protein 1 (*MX1*). The expression of *GBP1* and *IFIT1* was significantly higher in cells infected with RHDV2 in the absence of Rux than in Rux-treated cells; for *MX1*, the difference was not statistically significant (Fig. 3). Of note, the expression of *GBP1*, *IFIT1*, and *MX1* in control samples (mock-infected and infected for 1 h) was generally below the expression of the housekeeping gene *GAPDH* that was used for normalization. Our findings indicate that RHDV infection triggers the IFN response and that there is no constitutive expression of IFN-induced genes in uninfected cells.

### Rux enables serial RHDV passaging in monolayer cells

**Fig 4:**
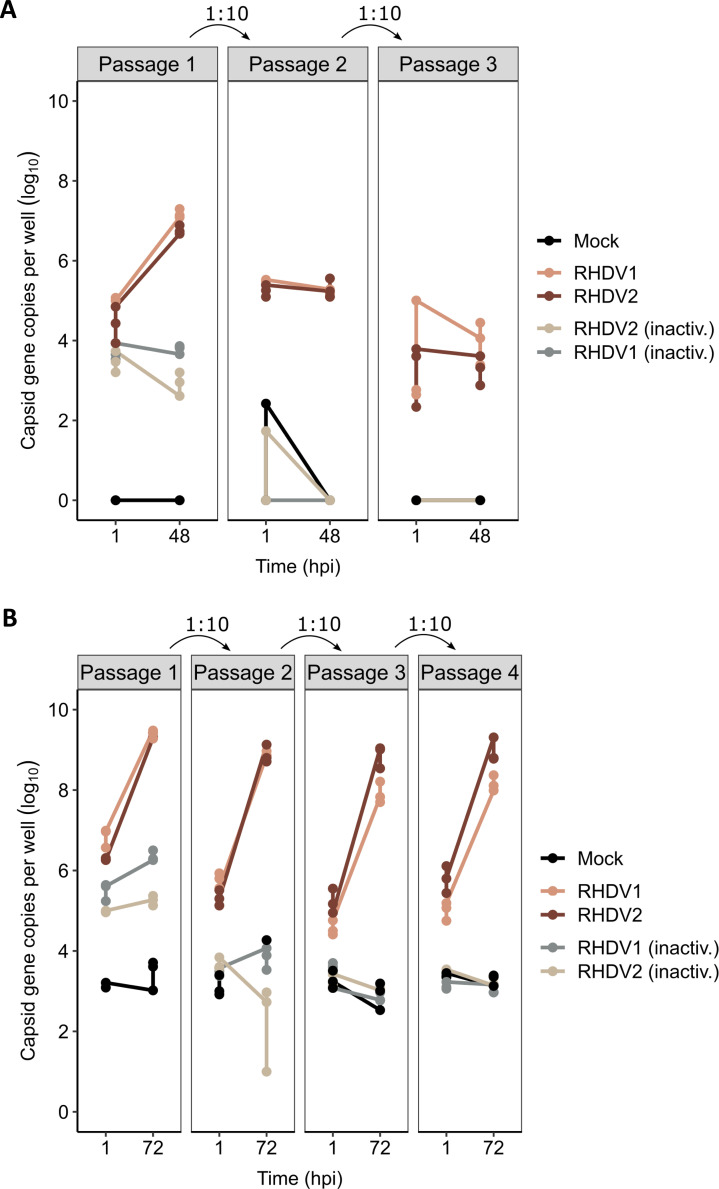
Passaging of RHDV in monolayer cells without Rux (**A**) and in the presence of 4 µM Rux (**B**). Cells were either mock-infected (Mock) or infected with 0.01 MOI of RHDV1, RHDV2, or heat-inactivated viruses (inactiv.). Capsid gene copy numbers were quantified at 1 and 48 (**A**) or 72 (**B**) hpi. The supernatant from passage 1 diluted at 1:10 was used as inoculum for passage 2 and so on until passage 3 (**A**) or 4 (**B**). Individual data points represent three biological replicates averaged from three technical replicates for each.

All previous attempts to serially passage RHDV in organoid-derived monolayers were not successful, despite an almost 2 log_10_ increase in virus titer during the first passage (a representative experiment is shown in Fig. 4A). We hypothesized that an activation of IFN-mediated antiviral pathways was responsible and tried passaging the virus in the presence of Rux. To that end, monolayer cells were inoculated with 0.01 MOI of either RHDV1, RHDV2, heat-inactivated viruses, or were mock-infected. Aliquots of supernatants were diluted 1:10 with fresh media and used as inoculum for the next passage; we performed a total of four passages and quantified the virus genome copy number for each passage at 1 and 72 hpi. In every passage, the titer in the supernatant almost reached the initial titer of the inoculum, demonstrating that the IFN inhibitor Rux allowed a productive passaging of both RHDV1 and RHDV2 (Fig. 4B).

**Fig 5:**
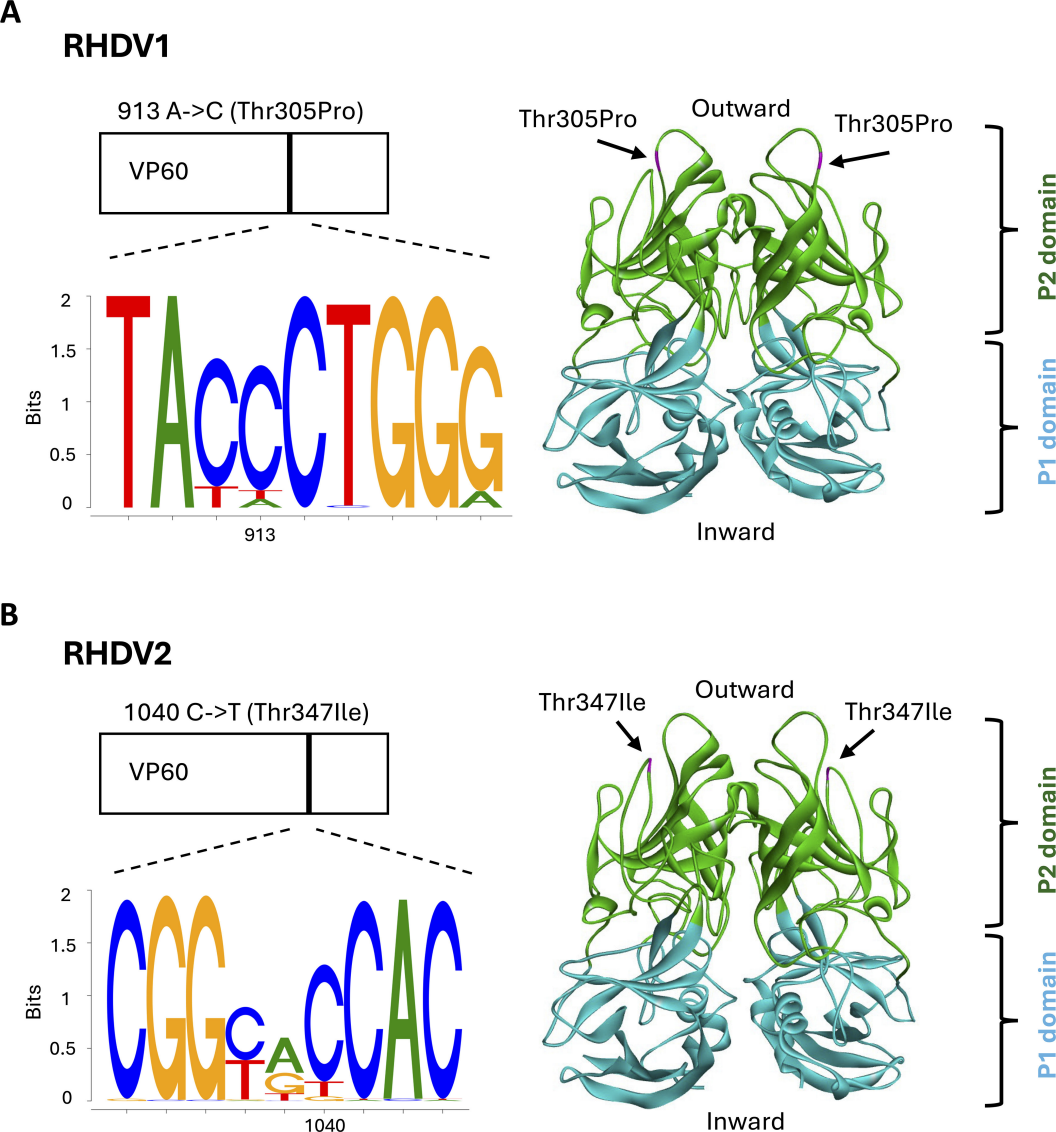
Changes to the RHDV1 and RHDV2 capsid gene (VP60) after serial passages in cell culture. Viruses were passaged in cell culture for four passages; inocula and supernatants from each passage were analyzed by sequencing. In RHDV1 (**A**), a variant at the position 913 was detected. In RHDV2 (**B**), a variant at the position 1,040 was detected. Both variants were present in the inocula at low frequencies but became dominant in the second passage. Sequence logos were generated using an alignment of RHDV1 (total of 100) or RHDV2 (total of 176) sequences and represent the occurrence of the detected variants in circulating strains. The structural model represents the protruding domain of a single VP60 dimer (PDB ID: 4X1X). The position of the corresponding amino acid change is highlighted in magenta and is pointed to by a black arrow; P1 and P2 domains are shown in blue and green, respectively. “Outward” and “inward” indicate the position of the capsid surface and the capsid base, respectively. The structural models shown in A and B are depicted at a slight angle to highlight the position of the changed amino acid in the respective variants. A, adenosine; C, cytosine; T, thymine; Ile, isoleucine; Pro, proline; Thr, threonine.

To determine whether passaging RHDV in cell culture selects for certain variants, the virus inocula and supernatants from each passage were analyzed by sequencing. For RHDV1 and RHDV2, we observed that a variant that was present in the respective inoculum at low frequency became more prevalent. For both viruses, the variation occurred in the protruding domain (P2) of the capsid protein (Fig. 5). In RHDV1, an adenosine to cytosine variant was detected at position 913, which resulted in an amino acid change from threonine to proline. Sequence analysis of 100 published RHDV1 sequences indicated that a cytosine at this position has higher occurrence than an adenosine (Fig. 5A). In RHDV2, a cytosine to a thymine variation at position 1,040 was detected that changed a threonine to isoleucine. Similarly, sequence analysis of RHDV2 sequences (total of 176) showed that both nucleotides occur at that position (Fig. 5B). Apart from these changes, we detected numerous other changes that appeared at either low frequency or in less than two replicates. A summary of all nucleotide changes detected in RHDV1 and RHDV2 genomes across passages is available in Fig. S1 and S2, respectively.

## DISCUSSION

The lack of a reliable culture system has long hampered research on rabbit caliciviruses. Our previously reported organoid culture system allows the growth of RHDV in cell culture, but virus infection was limited to a small number of cells (4). This led us to speculate that our undifferentiated organoid-derived monolayer cells are heterogeneous and that not all cells are susceptible to virus infection. Alternatively, the infection of permissive cells leads to the production of type I and/or III IFN, and IFN-induced proteins with antiviral activity inhibit the progression of the infection. To address the hypotheses, we first determined the cellular composition of organoid-derived monolayers using single-cell RNA sequencing and found that the monolayer cells consist of a homogenous population of immature cholangiocytes, confirming our previous observations obtained using marker antibody staining. This finding demonstrates that RHDV can infect more cell types than previously thought, adding cholangiocytes to the list of RHDV-permissive cells along with primary hepatocytes (42). Cholangiocytes form bile ducts that channel bile from the liver to the intestine (reviewed in reference 43). If the virus also replicates in the intestine, as was suggested previously (44), it would infect cells of the intestine before reaching the liver as it spreads via the fecal-oral route.

Next, we evaluated IFN responses in organoid-derived monolayer cells using the small molecule IFN inhibitor Rux. This and similar inhibitors have previously been shown to increase virus replication in both continuous and organoid-derived cells (6, 10–12). To this end, we tested the pan-IFN inhibitor Rux and observed that it significantly improved virus replication as measured by both RT-qPCR and immunofluorescence staining, indicating enhanced viral genome replication and translation, respectively. Notably, significant effects were observed after adding Rux to cell culture media as late as 1 h after the infection. A similar effect was observed in intestinal organoid monolayers that were infected with human norovirus (6). Taken together, Rux boosts the replication of difficult-to-cultivate caliciviruses in organoid-derived monolayer cells.

In addition to improved virus replication, we observed an unexpected effect of Rux on our organoid-derived monolayers. While previous studies reported that Rux can induce apoptosis and suppress the proliferation of cancer cells (13, 45, 46), we observed a beneficial effect of Rux on our organoid-derived monolayers, with cells appearing to grow faster and attach more strongly to the surface of culture flasks. This stabilizing effect is especially valuable for organoids and organoid-derived cells, as their growth characteristics can vary significantly, which affects the reproducibility of experiments (47). As we also observed variability in permissiveness between organoid cultures derived from different animals, we introduced routine batch-testing, that is, cells derived from each rabbit were tested for RHDV susceptibility using immunofluorescence staining of infected cells and RT-qPCR (as in Fig. 2G and H).

To analyze the effect of Rux on IFN-induced gene expression in our organoid-derived monolayer cells, we used RT-qPCR to quantify the expression of *MX1*, *IFIT1*, and *GBP1*. In the absence of an infection, these genes were expressed at base levels, while RHDV-infected cells expressed significantly higher levels. This indicates that RHDV triggers IFN responses and may explain why the addition of Rux shortly after infection was sufficient to improve virus replication. The expression of all three genes was higher in cells infected in the absence of Rux compared to cells cultured with Rux, which confirms that Rux inhibits IFN responses in our system. The products of *MX1*, *IFIT1*, and *GBP1* are proteins with antiviral activity that are usually expressed along with many other IFN-induced proteins. More work is needed to determine whether the expression of any of these proteins affects RHDV replication. Notably, *GBP1* expression is predominantly regulated by IFN-γ (type II IFN) (48), but its expression can also be induced by type I IFNs (49; reviewed in reference 50). Since our monolayer cells cannot express IFN-γ, the observed expression of *GBP1* was likely due to type I IFN signaling.

Although virus growth in rabbit organoids was observed previously without Rux (4), attempts to passage RHDV were not successful. The ability of Rux to inhibit IFN-mediated signaling and stabilize organoid-derived monolayers improved the productivity of our organoids to a point that allowed us to passage lagoviruses repeatedly. IFN inhibition is critical starting from the second passage, because the diluted supernatant contains IFNs induced during the first passage. The ability to productively grow and passage RHDV in cell culture will be useful for a range of applications, including the selection of improved biocontrol agents as well as the production of vaccines to protect pet and farmed rabbits. Previous studies generating a pipeline for the selection of a new rabbit biocontrol agent were resource-intensive and time-consuming, because they required the passaging of RHDV in passively immunized laboratory rabbits (51).

Sequence analysis of the viruses passaged in organoid-derived monolayer cells revealed several nucleotide changes that also occur in circulating strains. However, we cannot exclude that additional passages would result in viruses that replicate even better in cultured cells.

This work demonstrates that RHDV replication in rabbit liver organoid-derived monolayer cells can be improved by supplementing the cell culture medium with the IFN inhibitor Rux. The inhibition of IFN-induced innate immune responses allows repeated passaging of RHDV. Moreover, Rux supports cell growth and improves monolayer cell attachment. Overall, this study improves our understanding of the developed liver organoid-derived culture system and provides insights to increase its robustness.

## Data Availability

Genomic sequences of passaged RHDV1 and RHDV2 were submitted to GenBank (accession no. PV788770–95), and sequence reads were submitted to the Sequence Read Archive (accession no. PRJNA1276148).

## SUPPLEMENTAL MATERIAL

Figure S1 jvi.00574-25-s0001.tif Sequence analysis of the RHDV1 stock virus and progeny virus after passaging in rabbit organoid-derived monolayer cells.

Figure S2 jvi.00574-25-s0002.tif Sequence analysis of the RHDV2 stock virus and progeny virus after passaging in rabbit organoid-derived monolayer cells.

Table S1 jvi.00574-25-s0003.tif Primer sequences.

ASM does not own the copyrights to Supplemental Material that may be linked to, or accessed through, an article. The authors have granted ASM a non-exclusive, world-wide license to publish the Supplemental Material files. Please contact the corresponding author directly for reuse.
