# Supplementary figures and images for: Inhibition of interferon signaling improves rabbit calicivirus replication in biliary organoid cultures
Source: J Virol. 2025 Jul 25;99(8):e00574-25. doi: 10.1128/jvi.00574-25 (PMC12363232; doi:10.1128/jvi.00574-25)

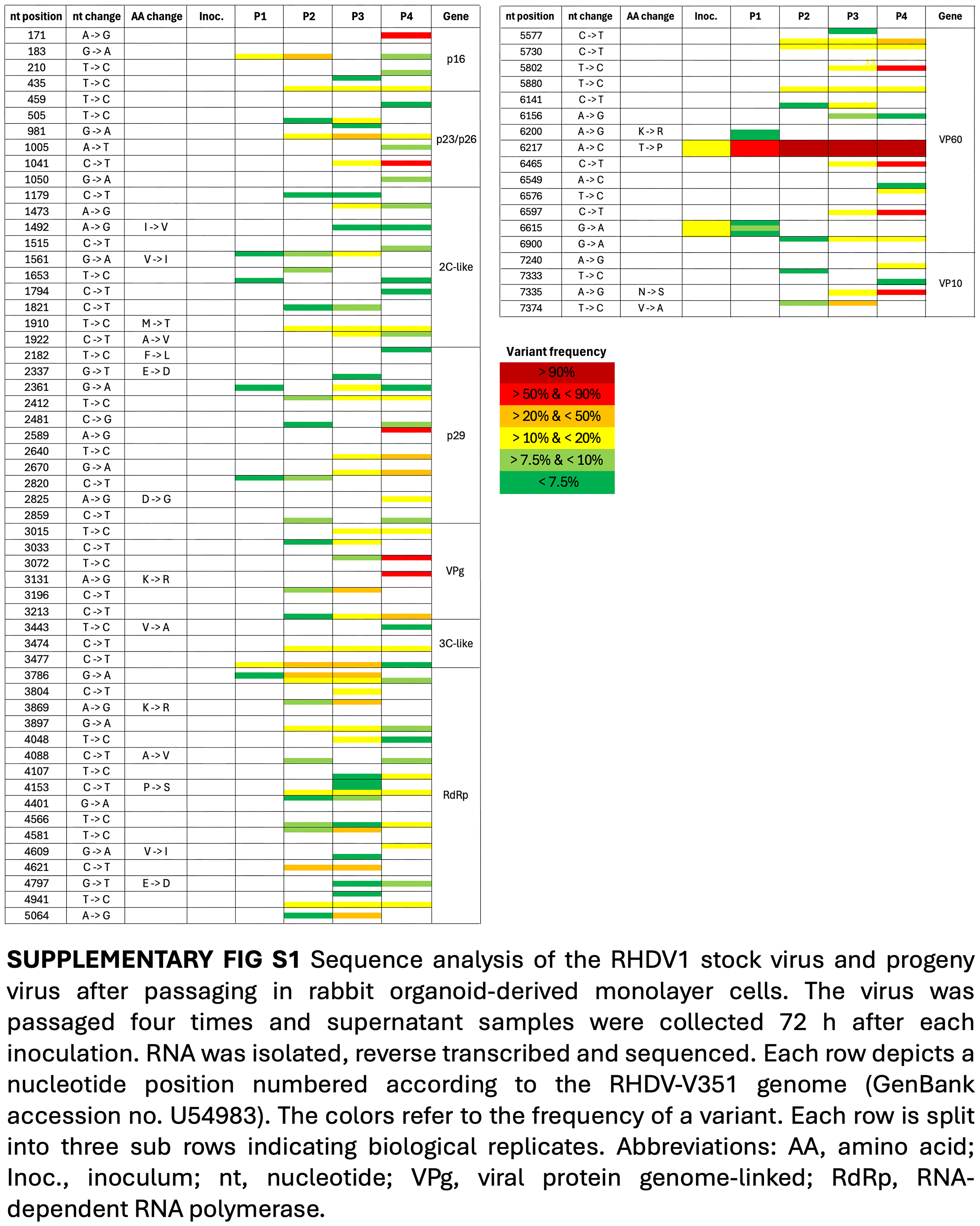

Supplement: Figure S1 — Sequence analysis of the RHDV1 stock virus and progeny virus after passaging in rabbit organoid-derived monolayer cells. [file jvi.00574-25-s0001.tif]

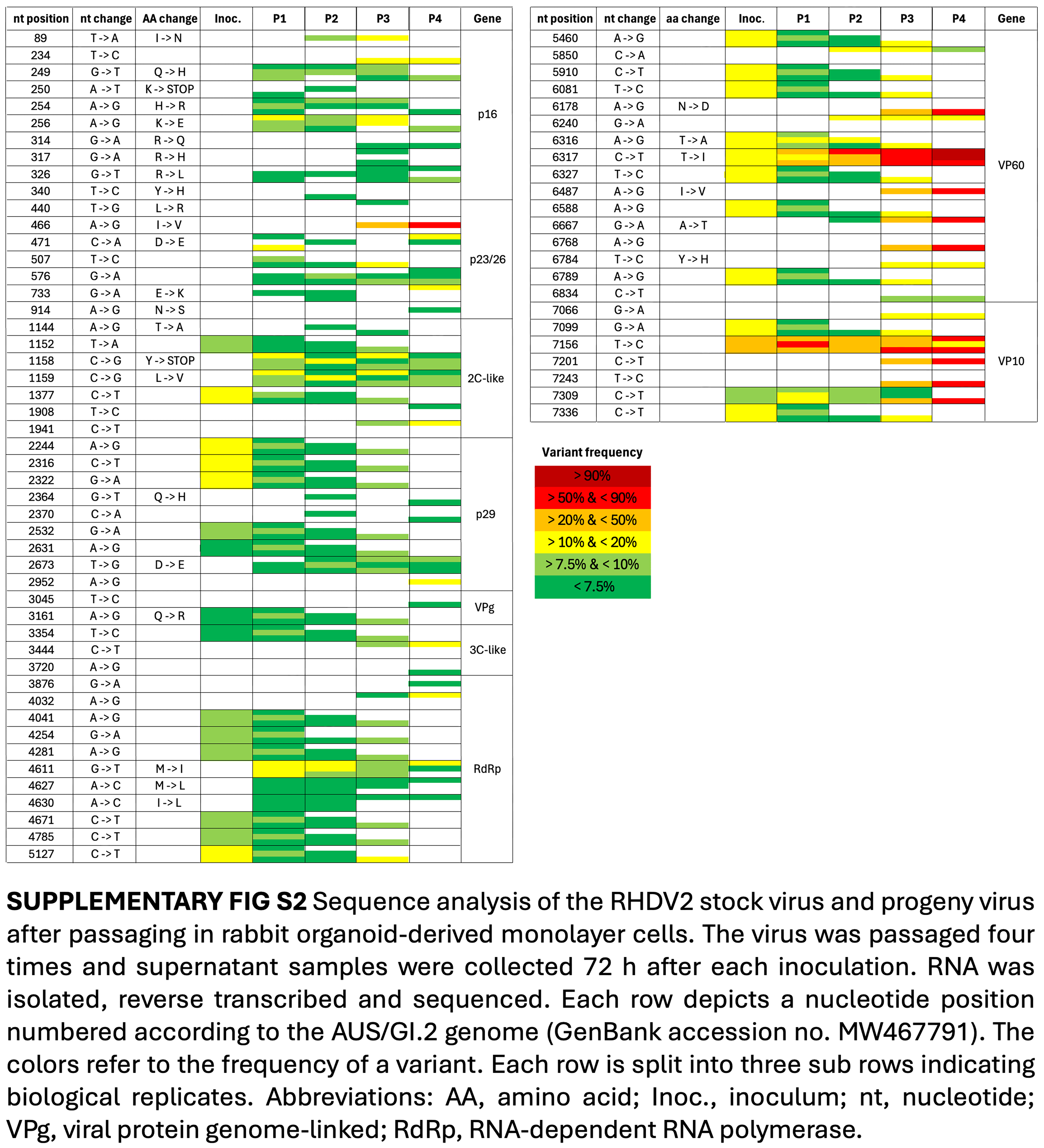

Supplement: Figure S2 — Sequence analysis of the RHDV2 stock virus and progeny virus after passaging in rabbit organoid-derived monolayer cells. [file jvi.00574-25-s0002.tif]

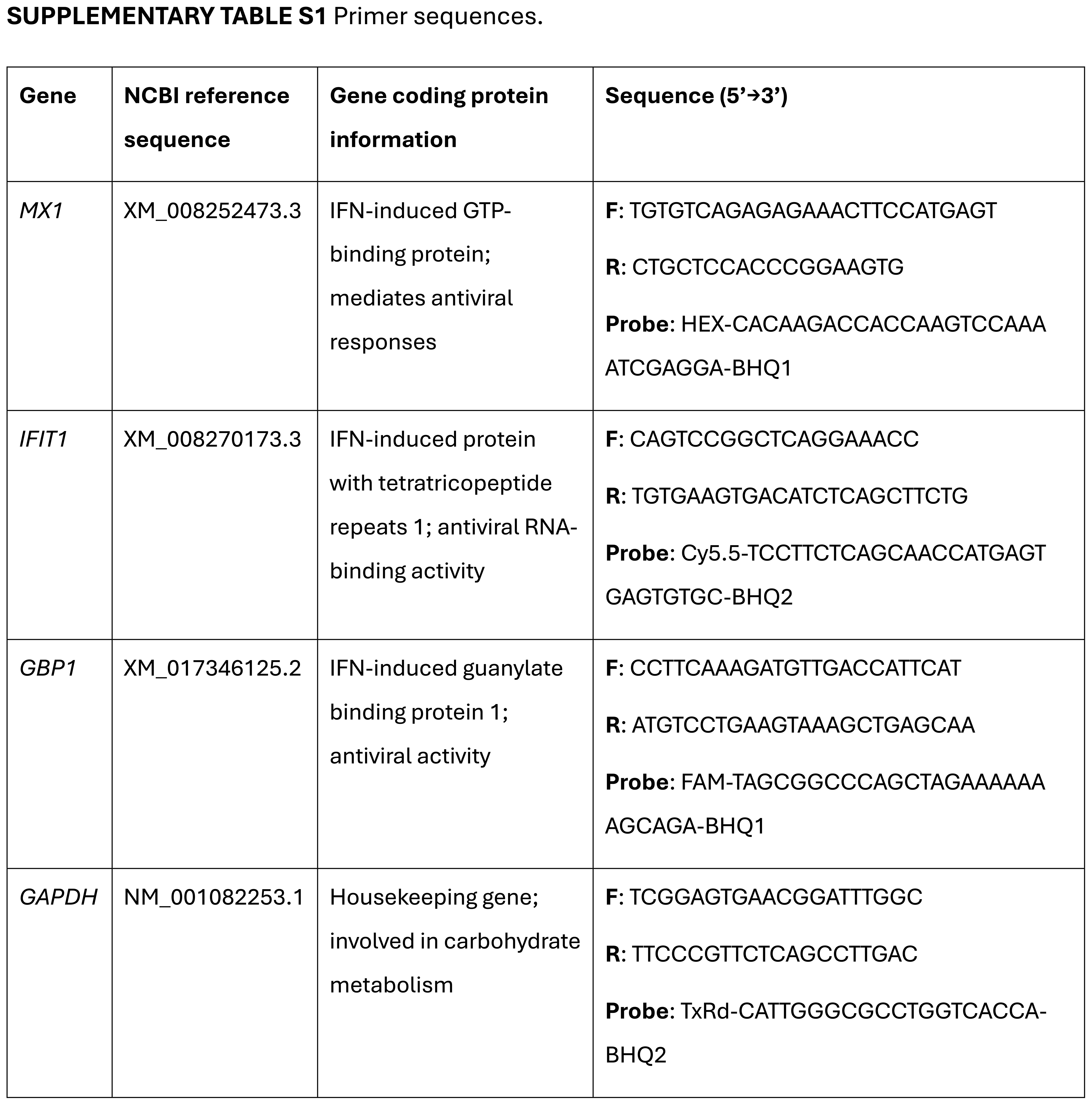

Supplement: Table S1 — Primer sequences. [file jvi.00574-25-s0003.tif]
